# Human papilloma virus circulating tumor DNA assay predicts treatment response in recurrent/metastatic head and neck squamous cell carcinoma

**DOI:** 10.18632/oncotarget.27992

**Published:** 2021-06-22

**Authors:** Catherine T. Haring, Chandan Bhambhani, Collin Brummel, Brittany Jewell, Emily Bellile, Molly E. Heft Neal, Erin Sandford, Ryan M. Spengler, Apurva Bhangale, Matthew E. Spector, Jonathan McHugh, Mark E. Prince, Michelle Mierzwa, Francis P. Worden, Muneesh Tewari, Paul L. Swiecicki, J. Chad Brenner

**Affiliations:** ^1^University of Michigan, Department of Otolaryngology-Head and Neck Surgery, Ann Arbor, MI 48109, USA; ^2^University of Michigan, Department of Internal Medicine, Division of Hematology and Oncology, Ann Arbor, MI 48109, USA; ^3^University of Michigan, Department of Biostatistics, Ann Arbor, MI 48109, USA; ^4^University of Michigan, Rogel Cancer Center, Ann Arbor, MI 48109, USA; ^5^University of Michigan, Department of Pathology, Ann Arbor, MI 48109, USA; ^6^University of Michigan, Department of Radiation Oncology, Ann Arbor, MI 48109, USA; ^7^University of Michigan, Department of Pharmacology, Ann Arbor, MI 48109, USA; ^8^University of Michigan, Department of Biomedical Engineering, Ann Arbor, MI 48109, USA; ^9^University of Michigan, Center for Computational Medicine and Bioinformatics, Ann Arbor, MI 48109, USA; ^#^Co-First Authors; ^*^Co-Senior Authors

**Keywords:** HPV, head and neck cancer, oropharyngeal cancer, ctDNA, circulating tumor DNA

## Abstract

Despite the rising incidence of human papillomavirus related (HPV+) oropharyngeal squamous cell carcinoma (OPSCC), treatment of metastatic disease remains palliative. Even with new treatments such as immunotherapy, response rates are low and can be delayed, while even mild tumor progression in the face of an ineffective therapy can lead to rapid death. Real-time biomarkers of response to therapy could improve outcomes by guiding early change of therapy in the metastatic setting. Herein, we developed and analytically validated a new droplet digital PCR (ddPCR)-based assay for HPV16 circulating tumor DNA (ctDNA) and evaluated plasma HPV16 ctDNA for predicting treatment response in metastatic HPV+ OPSCC. We found that longitudinal changes HPV16 ctDNA correlate with treatment response and that ctDNA responses are observed earlier than conventional imaging (average 70 days, range: 35–166). With additional validation in multi-site studies, this assay may enable early identification of treatment failure, allowing patients to be directed promptly toward clinical trials or alternative therapies.

## INTRODUCTION

Head and neck squamous cell carcinomas (HNSCC) constitute 3–5% of all malignancies worldwide and there are approximately 600,000 newly diagnosed cases annually [1, 2]. The majority of patients with HNSCC present with locally and/or regionally advanced disease at diagnosis. Despite the use of combined modality treatment, a significant proportion of patients develop unresectable recurrent or metastatic disease (R/M) HNSCC, for which treatment is palliative and consists of systemic therapy [3–5]. Even with the development of novel treatment regimens including immunotherapy, the median survival for patients with R/M HNSCC is typically less than one year [6]. There is an increasing incidence of human papillomavirus associated oropharyngeal squamous cell carcinoma (HPV+ OPSCC) and this represents one of only four cancers increasing in incidence in the United States [7].

A number of prognostic clinical factors have been identified for patients with R/M HNSCC including HPV status [8]. However, the only validated predictive biomarker is PD-L1, allowing for identification of patients who will have survival benefit with pembrolizumab versus chemotherapy as a first line treatment. Despite this, treatment response rates with currently available therapeutics remain low and no biomarker has been validated for dynamic response assessment or prediction of treatment benefit including PD-L1 [9–11]. Given this, efficacy of therapy can only able to be assessed after 9–12 weeks of treatment since radiographic responses are often delayed [6, 9, 11, 12]. As a result of the limited response rate, many patients experience significant progression leading to airway compromise and impaired functional status, rendering them unsuitable for further cancer directed therapy. A method is needed to rapidly identify those not benefiting from therapy, as well as to avoid progressive disease on a futile therapy and enable an earlier switch to a potentially efficacious treatment. A clear need exists for treatment individualization in R/M HNSCC, which necessitates identification of predictive biomarkers that can inform about response to therapy in real-time. A promising new biomarker that could meet these challenges, particularly identifying patients not benefiting from therapy prior to traditional imaging, is circulating tumor DNA (ctDNA).

Solid tumors are known to shed circulating tumor DNA (ctDNA) which is detectable throughout malignancies in numerous bodily fluids including saliva, urine, and plasma [13–15]. Plasma ctDNA analyses allow for non-invasive longitudinal monitoring of tumor specific genomic alterations which offers many potential benefits including assessment of treatment response in those undergoing systemic therapy [16, 17]. Numerous studies in metastatic cancers including those of the breast, colon, and non-small cell lung cancer have suggested that changes in ctDNA may predict radiographic response to therapy [18–20]. However, to date a comprehensive prospective analysis of ctDNA assay characteristics and predictive efficacy has not been performed in head and neck cancer.

Previous work in HPV+ OPSCC has demonstrated that plasma HPV16 ctDNA is detectable in the majority of patients with HPV-associated disease [14, 21, 22]. The most studied ctDNA biomarker in HPV+ OPSCC is HPV ctDNA. Limited prior data demonstrate that a rapid clearance profile of HPV ctDNA is associated with decreased risk of locoregional recurrence in patients receiving chemoradiation for locally advanced HPV+ OPSCC [23], and that levels may increase at the time of recurrence [21, 22, 24, 25]. Small studies have suggested that HPV ctDNA levels correlate with total disease burden and levels mirror fluctuations in disease status in patients with R/M HPV+ OPSCC [26]. However, no study has prospectively examined the potential of HPV ctDNA changes during the course of treatment to predict treatment outcome.

We hypothesized that: 1) a HPV16 ctDNA test would offer a precise assay for detection of HPV+ OPSCC and 2) the assay would predict progressive disease prior to radiographic imaging in patients undergoing treatment for R/M HPV+ OPSCC. We developed and analytically validated a highly sensitive and specific droplet digital PCR (ddPCR) assay for absolute quantification of HPV ctDNA from plasma specimens and performed an evaluation of clinical utility in a prospective, longitudinal patient cohort. Here we report details of assay development, analytical validation, and application to analysis of longitudinal clinical specimens from a R/M HPV+ OPSCC cohort undergoing systemic therapies.

## RESULTS

### Assay development and analytical validation

#### HPV16 ctDNA assay optimization

**Figure 1:**
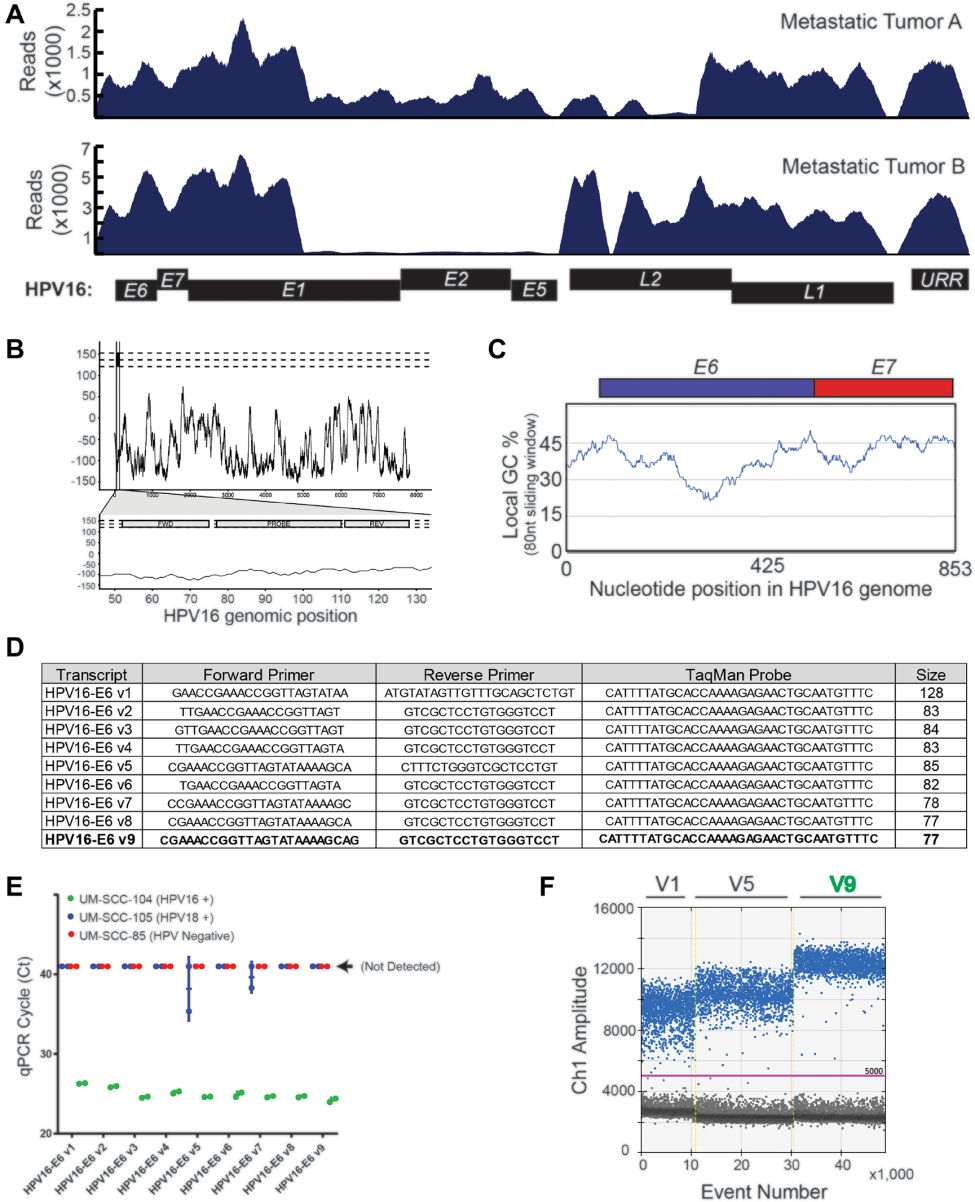
HPV16 plasma ctDNA assay development. (**A**) Targeted capture NGS data for the two HPV16+ R/M HNSCC patients with sufficient biopsy material for sequencing are shown. High density HPV16 probes were used for capture, and the absolute read mapping to the reference HPV genome is shown. (**B**) A continuous black line is plotted, representing the maximum “global-local” alignment score (y-axis) calculated between the HPV18 genomic sequence and each 1 nt-offset 18-mer sequence extracted from the HPV16 genome. The scores are plotted corresponding to the start position of each window. The three horizontal dashed lines indicate the maximum pairwise alignment score for the 18-mer sequence with 0, 2 or 4 “N” mutations randomly introduced into the 18-mer reverse primer sequence. The lower plot zooms in on the region encompassing the expected PCR amplicon, and the locations of the forward, reverse and probe annealing sites are indicated. (**C**) Percent of GC-content was calculated within an 80 nt sliding window. The genomic region of focus for assay design was plotted on the x-axis, such that the early region through nucleotide 853 (end of HPV16_E7) is shown. (**D**) Sequences of the nine different HPV16 primer-probe sets evaluated. (**E**) Results of amplification using each of the nine primer-probe sets on cell line genomic DNA (as indicated) was determined by quantitative, real-time PCR. UM-SCC-105, which is HPV18 positive was used as a specificity control, and UM-SCC-85, which is HPV negative, was used as a negative control. Ct value on the y-axis represents the cycle threshold value obtained in each case. (**F**) Three prioritized probes were evaluated on UM-SCC-104 cell line genomic DNA. Droplet generation, PCR, and droplet reading were performed and rain drop plots are shown.

To develop a novel HPV16 ctDNA assay, we first sought characterize regions of the HPV16 genome that are retained throughout the course of HPV+ HNSCC cancer progression. While the HPV E6 and E7 genes have been shown to be retained in lymph node metastases by *in situ* hybridization and/or PCR of E6/E7 [27, 28], to our knowledge a detailed characterization of the complete HPV genome in tumor metastases using a focused enrichment analysis has not yet been completed using sequencing-based approaches. Therefore, to provide comprehensive empirical evidence for regions of the HPV genome that are retained in metastatic lesions and could be the focus of our assay, we performed targeted capture based sequencing of biopsy specimens collected from our prospective patient cohort (Figure 1A). This confirmed that metastatic tumor genomes harbored genetic material from the upstream regulatory region (URR), E6/E7 and L1 loci, but also frequently lost genetic material that included a portion of E1, E2/E4, E8. Additionally, metastatic tumor A was also missing most of the L2 gene, supporting development of an assay within the L1 to E7 region of the genome.

In parallel, we implemented a bioinformatics-based approach to define local specificity of potential amplicon regions by comparing the HPV16 and HPV18 genomes. We chose these two genomes as they represent the highest and second highest HPV types in HPV+ cancers [29], respectively. We chose to use an 80 nucleotide sliding window to approximate the size of a small primer-probe amplicon-based assay (2 × 25 nt primers + 30 nt probe), given that most abundant cell free DNA expected to be accessible to standard ddPCR assay-design is in the range of 73–165 nts [30]. This demonstrated that the HPV16 and HPV18 genomes have minimal pairwise alignment at 80 nucleotide resolution with a few exceptions (Figure 1B). Consequently, we chose to focus assay design within the minimal region of HPV16 DNA retained in both tumor genomes from Figure 1A, excluding the beginning of the E1 gene which had a relatively high pairwise alignment between HPV16 and HPV18.

Finally, for optimal primer and probe design, we chose to focus our assay on a region of the HPV16 genome with GC-content around 45%, based on data showing the impact of GC-content on polymerase amplification ability of small amplicons [31]. Again using an 80nt sliding window, we identified three potential regions of the focus region for assay design (Figure 1C), and for initial design chose to focus on the region containing the end of the URR and beginning of E6 for our assay. Nine primer sets of varying lengths were designed to target this region with one assay focused on a 128 nucleotide amplicon and 8 assays closer to the minimal length of 83 to 77 nt (Figure 1D). Each set of primers/probes was tested on control cell line genomic DNA by qPCR to test the relative sensitivity of HPV16 detection (from HPV16+ UM-SCC-104 genomic DNA) and confirm specificity over the HPV18 and HPV negative genomes (using HPV18+ UM-SCC-105 and HPV- UM-SCC-85), Figure 1E. This demonstrated a high sensitivity of the short amplicon assays relative to the long amplicon assay, and also confirmed the specificity of HPV16 detection. As we intended to use the assay in a digital droplet PCR format, we then evaluated three of the probe sets (V1, V5 and V9) by ddPCR. As shown in the raindrop plots, assay V9 had the best signal-to-noise ratio and was therefore advanced for detailed analytical validation (Figure 1F).

#### Analytic validation of the HPV16 ctDNA ddPCR assay

**Figure 2:**
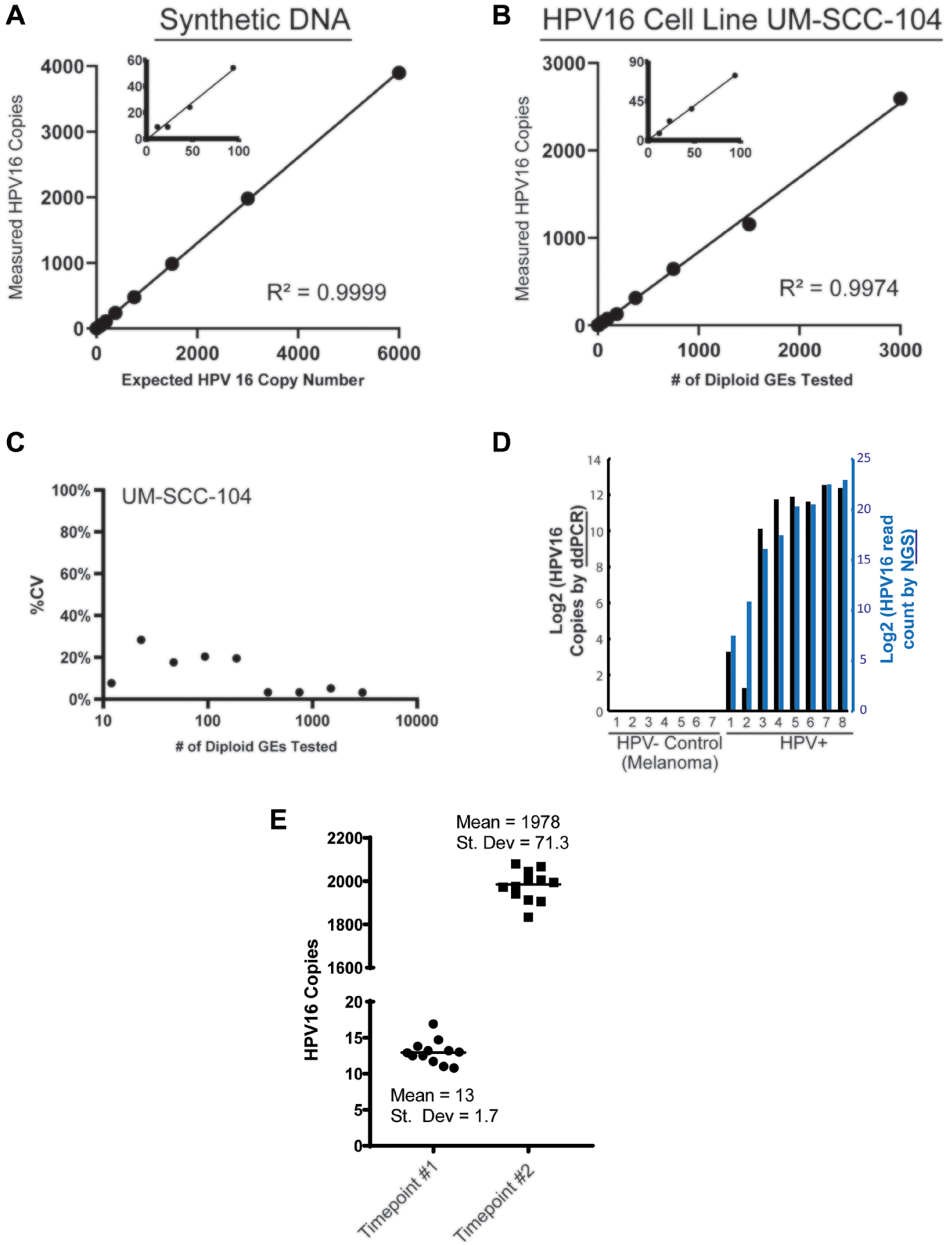
Analytical validation of HPV16 ctDNA droplet digital PCR assay. (**A**) Plot shows ddPCR assay results from a 2-fold dilution series (cumulative of 3 replicates) to determine the limit of detection (LOD) and reportable range of *HPV16 E6* ctDNA. The expected copies of a 87bp synthetic DNA fragment containing the 77bp amplicon region of the *HPV16* *E6* V9 assay, spiked into a human genomic DNA matrix (600,000 diploid GEs per data point) are shown on the *x-axis*, with the number of copies measured by the ddPCR analysis indicated on the *y-axis*. (**B**) The expected diploid GEs of *HPV16 E6* in UM-SCC-104 cell line DNA (*x-axis*) is plotted against the number of copies measured by the ddPCR a (*y-axis*; cumulative of 3 replicates). Based on the data shown, the LOD in both dilution series analyses (i.e., synthetic *HPV16* *E6* DNA fragment in (*A*) and UM-SCC-104 cell line DNA in (*B*)) was calculated to be < 5 copies per 20 uL reaction (see Materials and Methods). No positive droplets were observed when only *HindIII* digested human genomic DNA (200,000 diploid GEs per 20 ul reaction) was used as a non-HPV template (*n* = 15). (**C**) The plot shows % CV observed for *HPV16_E6* measured copy number at different dilutions plotted against the number of diploid GEs (converted to log_10_) of the UM-SCC-104 cell line DNA that was tested. (**D**) Tumor DNA extracted from FFPE-tumor specimens was analyzed with the HPV16 ddPCR assay. 7 melanomas were used as absolute HPV- samples and compared to 8 p16+ HNSCC tumors using the ddPCR assay (black bars), and compared to the HPV read counts from NGS (blue bars). (**E**) Multiple aliquots of plasma from one HPV16+ HNSCC patients obtained at two separate time points, with low and high ctDNA levels, were used for analysis of variance in the sample processing and analysis protocol.

To determine the limit of detection for the assay, we used serial dilutions of synthetic HPV16 DNA containing the 77bp amplicon specific to assay V9 (Figure 2A), as well as serial dilutions of UM-SCC-104 cell line genomic DNA (Figure 2B). Through genome sequencing analysis, UM-SCC-104 has previously been demonstrated to have low copy number of a near complete HPV16 genome [32]. The synthetic HPV16 fragment was tested in the context of a background of normal human genomic DNA used as a carrier. Both UMSCC-104 cell line genomic DNA and normal human carrier DNA were digested with HindIII restriction enzyme, to better recapitulate the fragmented nature of plasma cfDNA. The LoD by both dilution series experiments was determined to be < 5 copies per reaction (see Materials and Methods) (Figure 2A and 2B). The ddPCR assay displayed a low variation among replicates at different concentrations as seen in the coefficient of variation (CV) plot of the restriction digested UMSCC-104 cell line (Figure 2C), which is derived from an HPV16+ HNSCC.

To then confirm specificity of the ddPCR assay, we chose to compare assay results on genomic DNA isolated from a cohort p16+ OPSCC tumors to a series of melanoma tumor tissue specimens as HPV-negative controls. Importantly, this confirmed the high specificity of our ddPCR assay for HPV16+ tumor tissue (Figure 2C and 2D). Finally, to further characterize the reproducibility of the overall assay process, multiple aliquots of plasma from a single patient over two time points (one with a low level of HPV16 ctDNA and the other which had a high level) were isolated independently and run separately with our assay. For the low-count sample, the mean count was 13 copies/uL with a standard deviation of 1.7 copies/uL. For the high-count sample, the mean count was 1978 copies/uL with a standard deviation of 71.3 copies/uL (Figure 2D and 2E).

Collectively, this analytic data validated the high sensitivity and specificity of our HPV16 ddPCR assay.

### Clinical performance of HPV ctDNA assay

#### Patient characteristics

**Figure 3:**
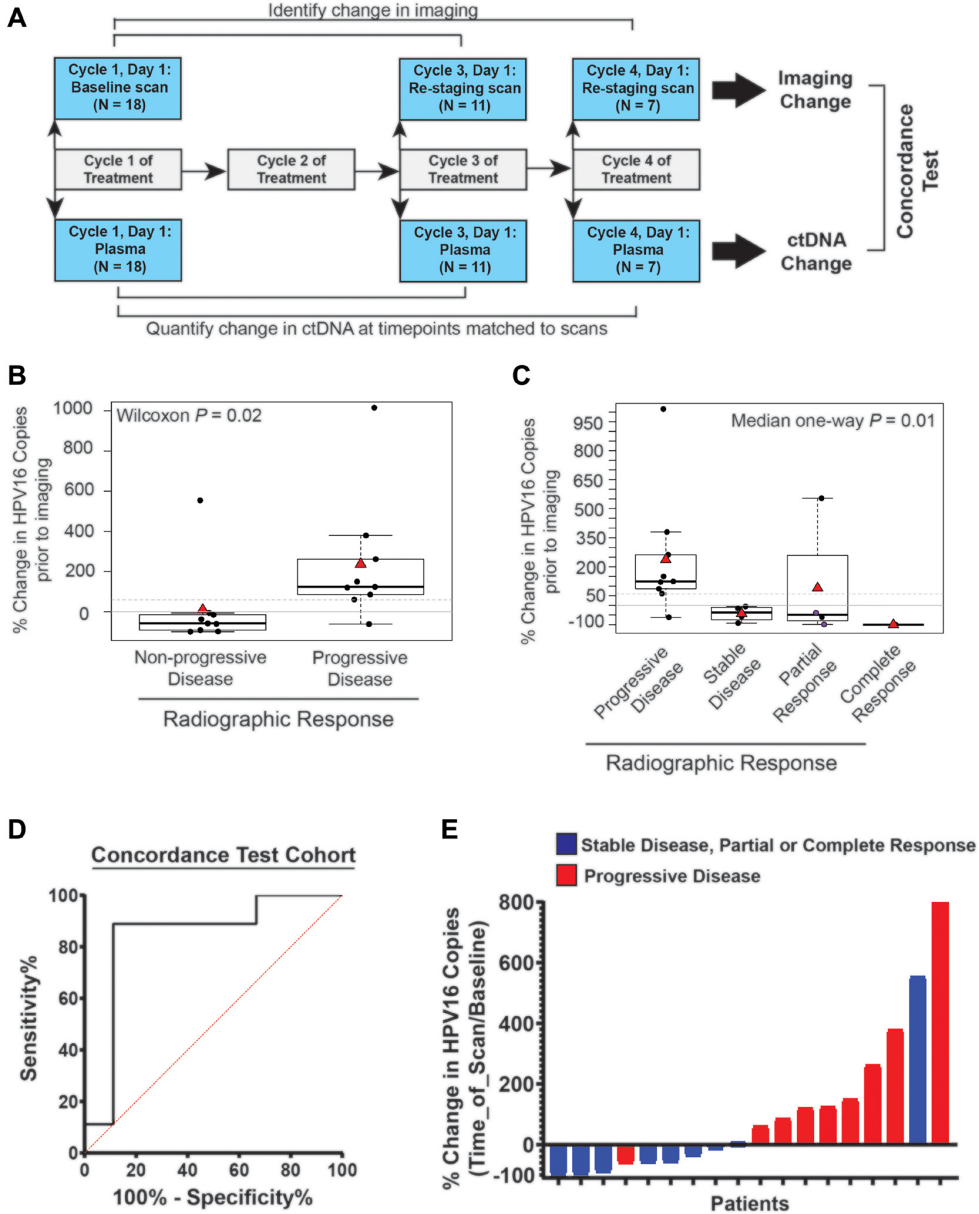
Time point matched changes in HPV16 ctDNA copies are highly concordant with changes in radiographic imaging in recurrent and metastatic HNSCC patients. (**A**) Schematic timeline representation of the cohort to evaluate the correlation of change in HPV16 ctDNA and change in imaging. The change in radiographic response between each patient’s baseline and re-staging CT scans (top bars) were compared to change in HPV16 ctDNA copies between a sample collected synchronously with imaging and a baseline sample (bottom bar). For this cohort, we were able to obtain plasma on imaging matched time points for 18 total treatment series. (**B**) Box-plot analysis. A Wilcoxon test was performed to assess the difference between percent change in HPV16 ctDNA in patients with progressive disease (PD) and those deriving benefit (non-PD) on restaging imaging. The dotted line indicates a 60% change as identified in ROC curve analysis. (**C**) Box-plot analysis. Median one-way analysis tests were performed to evaluate for differences in percent change of HPV ctDNA between patients with progressive disease (PD), stable disease (SD), partial response (PR), and complete response (CR) on restaging imaging. Median one-way analysis demonstrated statistically significant changes across response categories (*p* = 0.01). The dotted line indicates a 60% change as identified in ROC curve analysis. The two purple circles highlight patients whom were found to have pseudoprogression. (**D**) ROC curve analysis was performed and identified a ≥ 60% increase in HPV16 ctDNA being associated with the optimal sensitivity and specificity for predication of progression on radiographic imaging. (**E**) A waterfall plot demonstrates each patient’s percent change in HPV16 ctDNA level relative to baseline at the time of re-imaging. Color coding indicates radiographic response as indicated.

Sixteen patients with p16 positive tumors were enrolled between October 2017 and April 2019 in which we collected 102 distinct plasma samples. Our primers were able to detect HPV16 ctDNA in baseline samples from 12 of these patients (12/16, 75%), all of whom had HPV type 16. Baseline characteristics of the 12 subjects are summarized in Table 1. Blood was collected with Paxgene tubes for all time points in 10 patients and Streck Cell-Free BCT tubes for all time points in 2 patients. Of these 12 subjects, 6 had ctDNA levels analyzed during two distinct treatment courses representing a total of 18 distinct treatment courses (85 distinct samples). There was an average of 5 samples collected per treatment regimen (range: 2–17). Other than one patient missed plasma collection on Cycle 2, Day 1, there were no missing samples. Sample collection is outlined in Figure 3A. Seven patients were treated with immunotherapy-containing regimens, while 11 treatment courses utilized cytotoxic chemotherapy.

**Table 1:** Patient demographics and treatment characteristics

Patient Demographics (*n* = 12)	
Sex	
Male, *N* (%)	9 (75%)
Female, *N* (%)	3 (25%)
Age, years, Mean (SD)	62 (7.6)
Primary Treatment Received	
Chemoradiation, *N* (%)	11 (92%)
Surgery with adjuvant treatment as indicated, *N* (%)	1 (8%)
Smoking Status	
Current, *N* (%)	1 (8%)
Former, *N* (%)	3 (25%)
Never, *N* (%)	8 (67%)
Type of recurrence at R/M diagnosis	
Unresectable locoregional recurrence, *N* (%)	3 (25%)
Distant metastases, *N* (%)	9 (75%)
Treatments Characteristics (*n* = 18)^1^	
Chemotherapy, *N* (%)	11 (61%)
Immunotherapy, *N* (%)	7 (38%)
Timing of imaging and plasma collection	Average (range)
Restaging imaging after 2 cycles^2^	11
Restaging imaging after 3 cycles^3^	7
Baseline collection → restaging scans	70 d (35–166)
Baseline collection → Cycle 2 Day 1	27 d (20–49)
Cycle 2, Day 1→ restaging imaging	38 d (10–82)

^1^6 of 12 subjects had ctDNA collected during two distinct treatment courses representing a total of 18 distinct treatment courses. ^2^Performed on Cycle 3, Day 1. ^3^Performed on Cycle 4, Day 1.

Eleven patients had restaging imaging after two cycles whereas 7 patients had imaging after 3 cycles. The average duration from initiation of therapy (baseline plasma collection) to radiographic restaging imaging was 70 days (range: 33–166). The average duration of one cycle of treatment (baseline plasma collection to cycle 2 plasma collection) was 27 days (range: 21–44) and the average time from cycle 2 plasma collection to restaging scans was 43 days (range: 10–138) (Table 1).

#### Change in plasma HPV16 ctDNA levels correlates with radiographically-determined treatment response

We first assessed the concordance of detected radiographic progression at the time of standard of care re-imaging with an increase in HPV16 ctDNA observed in blood collected synchronously at the time of re-imaging. Thus, we evaluated the change in HPV16 ctDNA from baseline to the time of restaging imaging at post-cycle 2 therapy or post-cycle 3 therapy time points (Figure 3A). We found that percent change in HPV16 ctDNA was associated with radiographic progression (Figure 3B, Wilcoxon *p* = 0.02). Similarly, a relationship was observed for smaller increases (or larger decreases) in HPV16 ctDNA across response groups as defined by RECIST (PD, SD, PR, CR) (Figure 3C, Median analysis, *p* = 0.01). ROC curve analysis optimizing Youden’s index for prediction of progressive disease estimated a discriminatory cut point in the range of 30–60%. After evaluating the optimization in bootstrapped samples, we chose ≥ 60% increase as a cut-point for prediction of progressive disease with optimal sensitivity and specificity. Dichotomizing patients into those with ≥ 60% vs < 60% HPV16 ctDNA increase resulted in an AUC of 0.84 (*p* = 0.02 Figure 3D), sensitivity of 88.9% (95% CI 52-99.7%), specificity of 88.9% (95% CI 52–99.7%), positive predictive value of 88.9% (95% CI 55–98.1%) and negative predictive value of 88.9% (95% CI 56–98.1%). Of subjects with < 60% increase in HPV16 ctDNA, (8/9) had stable disease or partial response, and, of subjects with ≥ 60% increase in HPV16 ctDNA (8/9) had progressive disease (Figure 3E).

**Figure 4:**
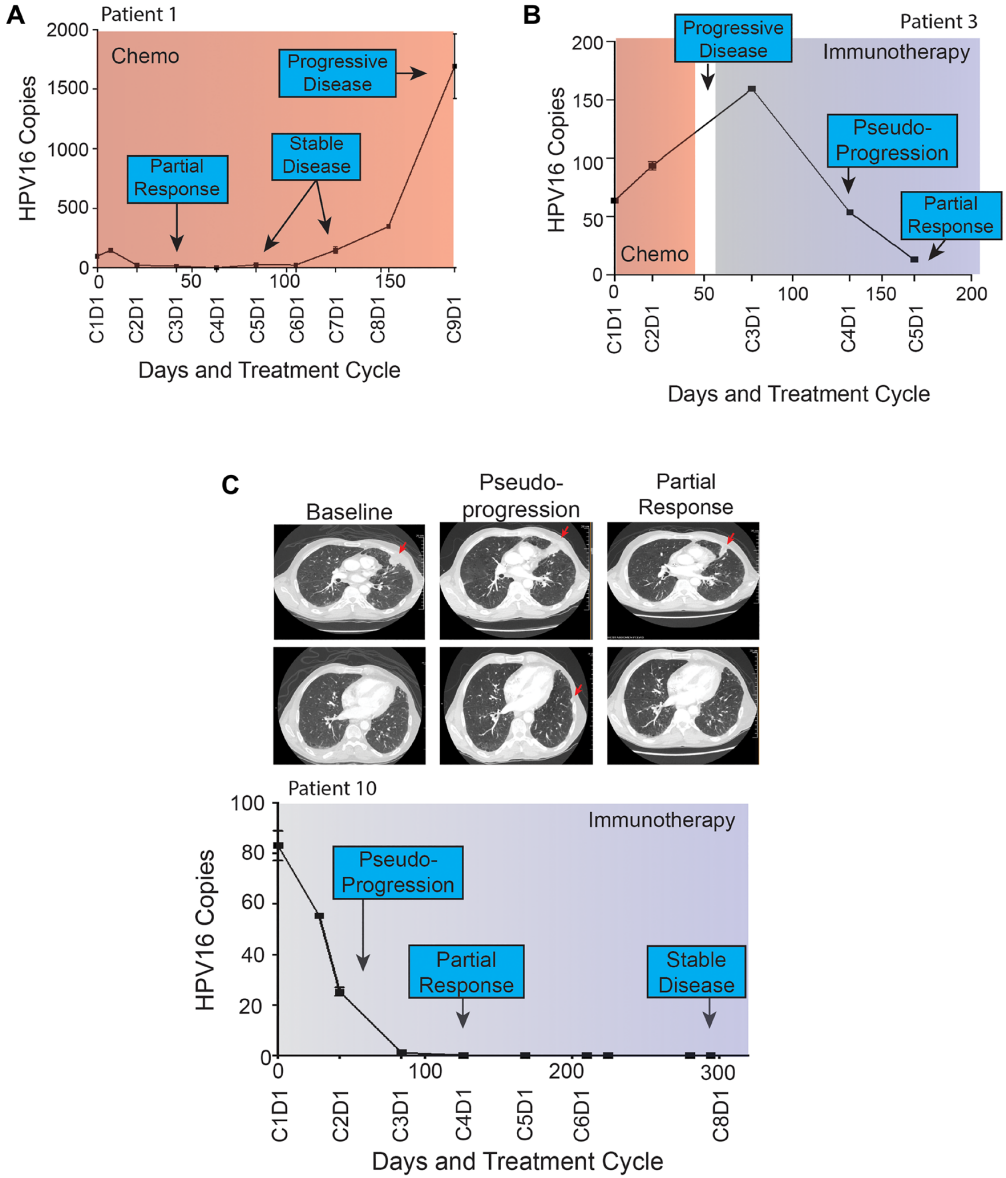
(**A**–**C**) Patient HPV16 ctDNA Levels and Treatment Histories. Plasma HPV16 ctDNA levels (copies per 1 μL) measured over time in patients with p16+ R/M HPV+ OPSCC (A–C). On the X-axis is days since study enrollment as well as cycle and day (CxDx) of treatment cycle. Colored boxes represent treatment courses (chemo = chemotherapy, immune = immunotherapy). Radiographic response are noted in each graph. Panel (A) highlights HPV16 ctDNA identifying progression more than 100 days prior to radiographic imaging. (B and C) highlight patients with radiographic pseudoprogression while treated with immunotherapy. Select serial CT images are shown for patient 10 at baseline, identification of pseudoprogression, and confirmatory imaging demonstrating partial response. A left pleural based soft tissue metastasis (top row) was initially noted to increase in size abutting the mediastinum, prior to decreasing in size dramatically. Similarly, a small sub-centimeter pleural based nodule (bottom row) initially grew to 1.1 cm prior to resolving completely on confirmatory imaging.

Next, we looked at the longitudinal pattern of HPV16 ctDNA within each patient in relationship to their clinical history including treatment modality, symptoms of progression, and imaging findings. A sample of patient HPV16 ctDNA levels and histories over time are shown in Figure 4. These graphs suggested that changes in HPV16 ctDNA levels occur prior to determination of radiographic response and an increase in ctDNA level precedes radiographic failures. This is remarkably shown in the case of Patient 1 (Figure 4A) in whom molecular progression was observed more than 100 days prior to detection of radiographic progression. Of particular interest were two cases of patients treated with immunotherapy in whom radiographic pseudo-progression was observed. In both cases, HPV16 ctDNA levels decreased, consistent with a molecular response despite suggestion of progressive disease on imaging (Figure 3C). Pseudoprogression has been described as an uncommon event occurring in less than 10% of patients treated with immunotherapy [33]. Patient 3 (Figure 4B) experienced potential symptoms of progression and initial response imaging was interpreted as progressive disease. Synchronous HPV16 ctDNA analysis noted a biochemical response with a 63% decrease. Treatment with immunotherapy was continued and the patient developed a partial response on follow up imaging which is ongoing for greater than 18 months. In a similar scenario, Patient 10 (depicted in Figure 4C) was treated with immunotherapy and the first set of restaging imaging demonstrated progressive disease as judged by RECIST. Given the possibility of pseudoprogression, he was continued on therapy and eventually achieved a durable near complete radiographic response until his death (unrelated to disease progression). HPV16 ctDNA analysis showed a marked decrease at the synchronous blood collection with 100% clearance of HPV16 ctDNA. Representative radiographic images from two different planes are shown in Figure 4C.

**Figure 5:**
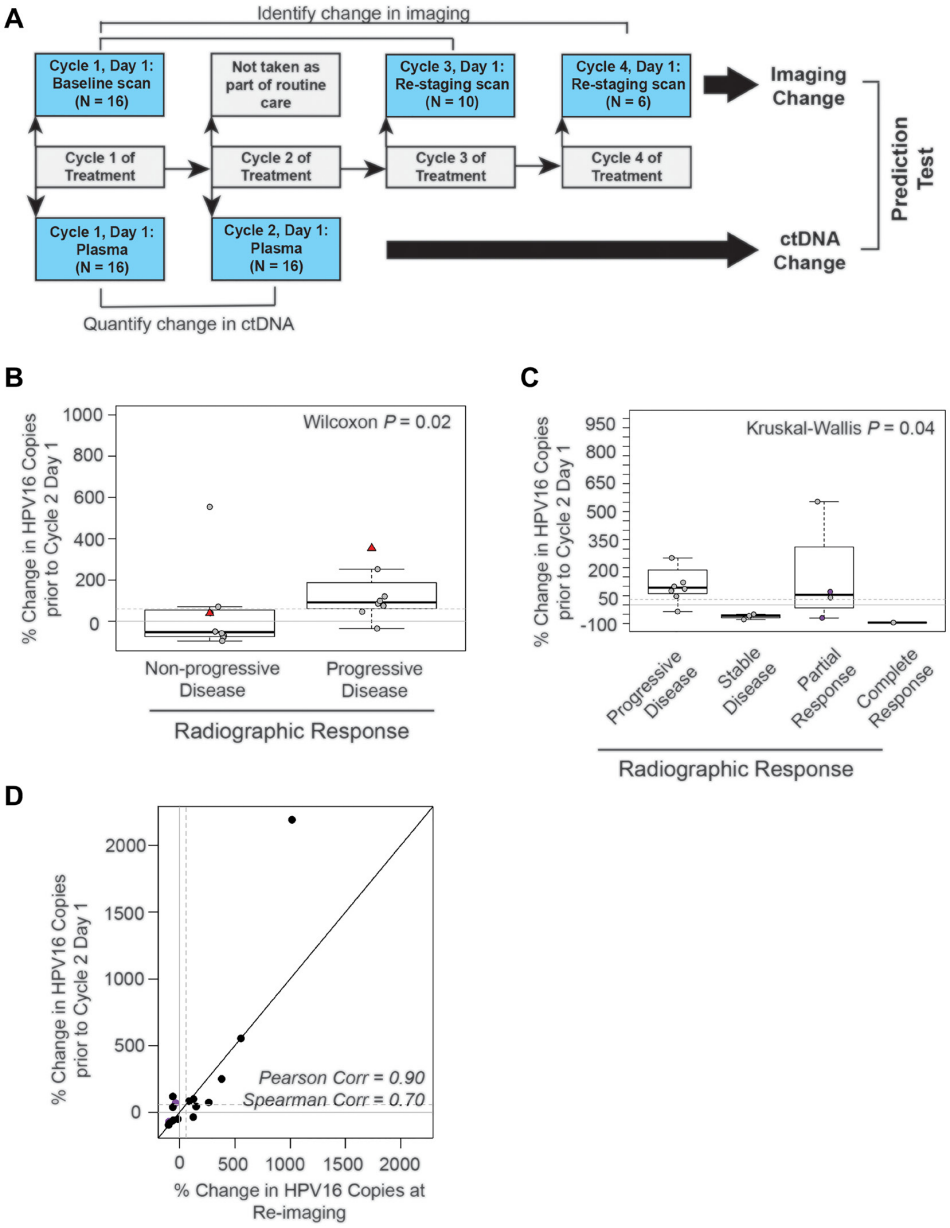
The absolute change in HPV16 ctDNA copies after one cycle of treatment predicts radiographic response in recurrent and metastatic HNSCC patients. (**A**) Schematic timeline representation of the design of the cohort to evaluate the predictive value of HPV16 ctDNA after one cycle of treatment. Change in radiographic response between each patient’s baseline and re-staging CT scans (top bars) were compared to change in HPV16 ctDNA copies between the post-cycle 1 of treatment time point and baseline (bottom bar). For this cohort, we were able to obtain plasma after the first cycle of treatment for 16 of the 18 potential treatment series as two patients missed blood collection; therefore, the total N analyzed is 16. (**B**) Box-plot analysis. Wilcoxon test was performed to assess the difference between percent change in HPV16 ctDNA after one cycle of treatment in patients with progressive disease (PD) and those deriving benefit (non-PD). The dotted line indicates a 60% change as identified in previous ROC curve analysis. (**C**) Box-plot analysis. The Kruskal-Wallis analysis test were performed to evaluate for differences in percent change of HPV ctDNA after one cycle of treatment between patients with progressive disease (PD), stable disease (SD), partial response (PR), and complete response (CR) on restaging imaging. Kruskal-Wallis test demonstrated statistically significant changes across response categories (*p* = 0.04). The dotted line indicates a 60% change. The purple circles highlight patients with pseudoprogression. (**D**) Scatter plot of percent change in HPV16 ctDNA observed in the draw after one cycle of treatment and the percent change in the blood drawn synchronous with restaging imaging. Spearman and Pearson’s rho is reported to assess the magnitude of correlation.

Given these observations, we assessed if early changes in HPV16 ctDNA were predictive of future radiographic response to therapy. To test this, we evaluated the change between baseline HPV16 ctDNA and that drawn prior to cycle 2 (Figures 5A and 3A). Sixteen subjects had available measurements after cycle 1 prior to cycle 2. The median (min-max) number of days prior to imaging was 33 (10–82). We found a significant association between change in HPV16 ctDNA at cycle 2 and later evidence of progression (Figure 5B, Wilcoxon *p* = 0.02). A relationship was similarly observed for changes in HPV16 ctDNA across RECIST response categories which was statistically significant (Figure 5C, Kruskal-Wallis *p* = 0.04). There was strong positive correlation between percent change seen in the draw prior to cycle 2 to that drawn synchronous to re-staging imaging (Figure 5D, Pearson rho = 0.90).

## DISCUSSION

We described our development and detailed analytical validation of a high-precision ddPCR assay to quantify plasma HPV16 ctDNA and demonstrated through a prospective study that results of radiographic assessment and simultaneously collected HPV16 ctDNA were highly concordant. Furthermore, analysis suggests change in HPV16 ctDNA after just one cycle of treatment may be predictive of radiographic progression. Specifically, our data suggests that a < 60% increase (or any decrease) in HPV16 ctDNA is associated with a favorable response to therapy (complete response, partial response, or stable disease), whereas patients with ≥ 60% increase in HPV16 ctDNA have disease progression and presumably do not derive any benefit from therapy. Finally, these data demonstrate that the percent change in HPV16 ctDNA after a single cycle of treatment correlates with that obtained synchronous to re-staging imaging.

Despite introduction of immunotherapeutic agents, survival for patients with R/M HNSCC remains poor [6]. Initial response to therapy can have a significant favorable impact on survival for some patients [34]. however we do not have a clear understanding of which patients will respond to each therapy. Additionally, futile systemic treatments can have significant toxic side effects. Currently established tissue biomarkers, including PD-L1, could theoretically be used to guide initial treatment selection based on historical outcomes but require invasive biopsy. In OPSCC, biopsies are often challenging due to the nature of the anatomy involved. Circulating biomarkers, on the other hand, allow for dynamic quantification of real-time treatment response obtained via minimally invasive approaches. The use of plasma ctDNA to guide treatment decisions is increasing in the oncology clinical setting [35]. For example, PCR-based ctDNA assays for EGFR genotyping in non-small cell lung cancer, and for KRAS genotyping in colorectal cancer have demonstrated clinical validity and thus have received regulatory approval in the United States and Europe [36–38]. Changes in HPV16 ctDNA prior to radiographic responses have been characterized in locally advanced HPV+ OPSCC, however little investigation has been performed to date in R/M HPV+ OPSCC. A previous study in R/M HPV+ OPSCC suggested an association between HPV16 ctDNA and patient characteristics in HPV+ OPSCC [26]. This work demonstrated that patients with poor outcomes had higher plasma levels of ctDNA and that patients who responded to treatment were seen to have decrease in plasma HPV16 ctDNA levels whereas patients with progressive disease had increases in plasma HPV16 ctDNA. However, this was in a 17 patient cohort of which patients were enrolled either on active surveillance or already undergoing therapy and blood was collected on a rolling basis rather than protocol defined time points. Hence, although provocative, interpretation is limited given the heterogeneity of treatment status at the time of enrollment and timings of sample collection.

Our data suggests a potential role for HPV16 ctDNA for early detection of progressive disease in R/M HPV+ OPSCC. If validated as a predictive biomarker, this assay could be a key tool in guiding treatment decisions regarding efficacy of therapy, thereby potentially switching patients from futile therapy to more efficacious therapy more promptly. Furthermore, HPV16 ctDNA may serve as a tool to distinguish clinical benefit and progressive disease on imaging in patients treated with immunotherapy given the phenomenon of pseudo-progression. Although large-scale prospective studies are required to further test these hypotheses, one could envision a paradigm-defining trial in head and neck cancer in which systemic therapy is switched after one cycle based on biomarker indications of treatment failure, rather than delaying that intervention until radiographic imaging demonstrates disease progression.

This study has important limitations. When applied to our prospective cohort, our primers detected HPV16 ctDNA in 75% (12/16) of patients with HPV+ OPSCC (as determined by tumor p16 immunohistochemistry). There are many biologic factors that may affect HPV concordance between tumor and plasma. One possibility is that some tumors are simply not shedding ample HPV16 ctDNA for detection due to their size, rate of growth, or location of metastatic deposits. Although further assay development could help, this level of clinical sensitivity is in keeping with approved ctDNA assays used routinely in clinical practice. These assays, including EGFR ctDNA assay in non-small cell lung cancer, have been validated for clinical use and demonstrate the concordance of mutation detection between the primary tumor tissue and plasma ranges from 70–90% [39–41] Previous studies in HPV+ OPSCC have shown detected HPV16 ctDNA at similar frequencies [21–23]. Future assay modifications have the potential to improve the sensitivity of the assay. Changes in the region of the probe to change or improve coverage of the HPV16 genome may result in greater sensitivity to detect HPV+ OPSCC due to HPV16. As a minority of HPV+ OPSCC are caused by non-HPV16 strains, adding detection of other high risk HPV strains (i.e., 18, 31, 33) may improve the sensitivity. Further research is required to define the impact of these technical refinements. As aforementioned, this pilot cohort was utilized to assess for potential signal of clinical utility. While our data describes the diagnostic performance of an HPV16 ctDNA assay and suggest promise for the use of this assay to predict progressive disease in patients with R/M HNSCC, our data cannot establish clinical validity or utility. These data merit further investigation with validation in an appropriately powered, large uniformly treated cohort. Validation of candidate biomarkers requires multiple independent patient cohorts prior to clinical evaluation for utility, in which biomarker status is used to guide clinical management [42–44].

This data suggests that changes in HPV16 ctDNA may be predictive of progressive disease in patients with R/M HNSCC receiving systemic therapy. Changes in HPV16 ctDNA appear to precede radiographic response and thus have the potential to be used as an early predictive biomarker to guide treatment decisions, potentially improving survival and sparing toxicity. While larger prospective studies are required for validation and clinical utility analyses, this data offers a promising glimpse into the future potential clinical utility of HPV16 ctDNA in HNSCC.

## MATERIALS AND METHODS

### Development of HPV ctDNA assay

#### Droplet digital PCR (ddPCR) assay for HPV16 ctDNA

We developed a Taqman probe-based ddPCR assay for the most common HPV subtype, HPV16, as described below. Each ddPCR sample reaction contained 7 uL of DNA with 1 uL HPV target assay mix (forward and reverse primers and TaqMan FAM probe), 1 uL RPP30 assay mix (with HEX probe, Assay ID: dHsaCP2500350), 1 uL nuclease free water, and 10 uL ddPCR Super Mix (no dUTP) (#186-3024, Bio-Rad, Hercules, CA, USA). A ddPCR assay for the RPP30 control gene was used to assess sample quality (Bio-Rad, Hercules, CA, USA). Each sample assay was run in duplicate. UM-SCC-104 (HPV16 positive) and UM-SCC-105 (HPV18 positive) cell line DNA were run to serve as positive and negative controls.

Droplet generation was performed using the QX200 Droplet Digital PCR System (Bio-Rad, Hercules, CA). PCR was run with the following thermocycler conditions: enzyme activation for 10 minutes at 95°C for 1 cycle, denaturation at 94°C for 30 seconds and annealing/extension at 60°C for 60 seconds for 40 cycles at a 2°C/second ramp rate, enzyme deactivation for 10 minutes at 98°C for 1 cycle, followed by a hold at 4°C. Droplets were read using QuantaSoft Software (Bio-Rad, Hercules, CA, USA). Standardized thresholds for positive and negative droplets were used across samples. Samples were considered positive if they had 2 or more positive droplets at the same amplitude as the positive cell line control. The Bio-Rad system quantifies DNA in copies per uL of the 20 uL PCR reaction, which are the values reported here.

#### Calculation of limit of detection and coefficient of variation

To determine the Limit of detection (LoD), a synthetic 87 bp dsDNA fragment containing the 77 bp *HPV16 E6* amplicon from assay V9 (5′ AATGCCGAAACCGGTTAGTATAAAAGCAGACATTTTATGCACCAAAAGAGAACTGCAATGTTTCAGGACCCACAGGAGCGACTCACT 3′) or genomic DNA from UM-SCC-104 cell line was tested in a background human genomic DNA matrix (200,000 genome equivalents (GEs) per 20 μl ddPCR reaction). In order to generate background matrix DNA, human genomic DNA (#PR-G3041, Promega) was incubated with 1 unit HindIII enzyme (# R0104S, New England Biolabs) per microgram of DNA at 37°C for 4 hours followed by enzyme inactivation at 80°C for 20 min. A 2-fold dilution series (2000 copies or GEs per μl to 4 copies or GEs per ul) was tested in triplicates using ddPCR and LoD was calculated using the formula:

LoD = Limit of Blank (LoB) + 1.645 (Standard Deviation _low concentration sample_)

LoB was determined per 20 uL reaction using 200,000 diploid human GEs (HindIII digested) as a non-HPV background matrix (*n* = 15). With UM-SCC-104 cell line, LoD = 0.3 copies/20 μl reaction based on standard deviation of 0.2 for the lowest concentration sample (4 GEs/μl); and with synthetic DNA (containing the 77bp amplicon sequence), LoD = 4.2 copies/20 μl reaction based on standard deviation of 2.54 for the lowest concentration sample (4 copies/μl). The Coefficient of Variation (CV) was calculated using the formula % CV = (Standard Deviation/Mean) × 100.

#### Characterizing the precision of the HPV16 ctDNA ddPCR assay

One HPV16 ctDNA sample with low counts and one sample with high counts were selected to run reproducibility testing. ctDNA was extracted from 3 × 2 mL aliquots and each diluted 1:40 as described below. From this, 12 reaction assays were created for each sample. Droplet generation, PCR amplification, and droplet reading were performed as indicated above. HPV16 ctDNA counts were plotted. Means with standard deviations were calculated for the low and high sample. Standard curves were created to determine expected standard deviations for any given HPV16 ctDNA count.

#### HPV16 genome specificity analysis

The genomic sequence for HPV16 (K02718.1) and HPV18 (AY262282.1) were downloaded from Genbank. One nucleotide-offset sliding windows of 18, 24, 34 and 77 nt were extracted from the HPV16 reference sequence, corresponding to the lengths of the reverse primer, forward primer, probe and amplicon sequence, respectively. Pairwise alignment scores were calculated between each of the HPV16 k-mer window sequences and the HPV18 genome using the pairwise alignment function (R package, Biostrings v2.52.0) with default settings for the “global-local” alignment type and scoreOnly = TRUE, which returns the maximum alignment score for each k-mer window sequence. The maximum alignment score for each k-mer window was plotted based on the start position of each window. Using the same settings, pairwise alignment scores were also calculated between the HPV18 genome and the HPV16 primer, probe and amplicon sequences, with 0-4 mutations (substituting “N” bases) introduced at random locations. Plots were generated using the R packages, ggplot2 (v3.2.1), cowplot (v1.0.0) and ggforce (v0.3.2).

### Evaluation of assay performance in clinical cohort

#### Prospective cohort

A prospective cohort was enrolled as part of a University of Michigan Institutional Review Board-approved, observational longitudinal biospecimen collection study (UM IRB 00042189). All patients provided written informed consent. Patients ≥ 18 years old with histologically documented HPV+ OPSCC undergoing systemic therapy were eligible. p16 was determined by tumor immunohistochemistry and was used as a surrogate marker for HPV status of a patient’s cancer. All patients were required to have the presence of measurable disease by CT scan, or cutaneous lesions ≥ 10 mm not assessable on imaging but present on physical exam. Adequate hematopoietic and renal function were required and defined as hemoglobin ≥ 9.0 g/dL and serum

Enrolled patients had baseline collection of clinical data on demographics, disease characteristics, treatment plan, laboratory studies (CBC with differential, comprehensive metabolic profile) and radiographic staging studies. Pre-treatment tumor tissue was obtained for next generation sequencing, whenever available. Duration of a treatment cycle was defined based on clinical documentation. Blood was collected at each visit during the treatment course as well as synchronous with radiographic imaging until disease progression, patient withdrawal of consent, or at investigator discretion. 10 mL of blood was collected from patients in sterile PreAnalytix Paxgene tubes (PreAnalytiX GmbH, Switzerland) or Streck Cell-Free DNA BCT tubes (Streck Inc., Omaha, NE, USA) with the same type of tube used for serial collection within a given participant. Previous publications of ctDNA analysis have demonstrated equivalence between these collection tubes [45]. Tubes were held at room temperature and processed as per manufacturer’s recommendations within 7 days of collections as described in detail below. Re-staging imaging (after 2 or 3 cycles) was obtained at the frequency as determined by the discretion of the treating physician. Timing of the imaging and corresponding results were recorded in the database. Radiologic response was assessed according to RECIST v1.1. In the case of treatment continued despite RECIST-assessed progressive disease, due to suspected pseudoprogression, response was confirmed on subsequent scans. If a patient experienced progression and was started on a subsequent therapy, they were re-enrolled with identical approach of data and specimen collection.

#### DNA isolation from tumor tissue

Two tumor tissue blocks from patients with R/M HPV+ OPSCC from our prospective cohort were available with sufficient material for NGS and eight blocks from primary HPV+ OPSCC and 7 with melanoma blocks were available with sufficient material for ddPCR based tumor analysis. DNA extraction from FFPE specimens was performed as previously described [1, 2]. Areas of tumor were identified by a board-certified head and neck pathologist (J.B.M). Tumor and adjacent normal tissue cores were collected and genomic DNA was obtained using a Qiagen Allprep DNA/RNA FFPE kit (Qiagen, Hilden, Germany) and quantified using Qubit as previously described [46]. For ddPCR assays, RPP30 Bio-Rad assay was used to measure RPP30 reference gene and ensure ample DNA being assessed in the case of HPV positive and HPV negative tumors (data not shown).

#### Targeted next-generation sequencing to define HPV16 tumor DNA content

DNA from tumor tissue was submitted to the University of Michigan Advanced Genomics Core for targeted capture sequencing using the DNA Thruplex kit (Takara Biosciences). Targeted capture was performed using a custom designed probe panel from Nextera with high density coverage of HPV16 which is described in O’Leary et. al 2020. [19] Following library preparation and capture, the samples were sequenced on an Illumina HiSEQ4000, respectively, with paired-end 150 nt reads. Data was de-multiplexed and FastQ files were generated. Samtools depth (samtools/1.9) was used to compute the read depth at each position across the HPV16 genome. The input is a BAM file derived from HPViewer with short reads aligned to the HPV genome. The unmapped, secondary, QC-fail and duplicate reads were skipped from the analysis. Only reads with base quality greater than 5 and mapping quality greater than 20 were counted, plots were created with Excel.

#### DNA extraction from plasma

Plasma was isolated in two sequential centrifugation steps per manufacturer recommendations. Briefly, PaxGene tubes were centrifuged at 1900×g for 15 minutes; plasma layer was transferred into a 15 mL conical tube and centrifuged a second time again at 1900×g for 10 minutes. Streck tubes were centrifuged at 300×g for 20 minutes; plasma layer was transferred into a 15 mL conical tube and centrifuged a second time at 4600×g (maximum possible speed) for 10 minutes. Cell free DNA was isolated from a 2 mL aliquot of plasma using QIAamp^®^ MiniElute^®^ ccfDNA Mini Kit (Qiagen, Germantown, MD, USA) and diluted 1:40 according to manufacturer instructions. Genomic DNA from cell lines UM-SCC-104 (HPV16 positive), UM-SCC-105 (HPV18 positive), and UM-SCC-85 (HPV negative) [47] was extracted using the Promega Wizard DNA Purification Kit protocol (Promega A1120, Madison WI, USA). Cell line DNA was used for assay optimization and as positive controls for plasma ctDNA assays.

#### Statistical analyses

The primary aim of the prospective pilot cohort was to assess the HPV16 ctDNA assay in a diverse patient population and assess for 1) concordance with imaging results and 2) potential signal of association of early dynamics with radiographic progression. Treatment responses, as determined by radiology, after 2-3 cycles of treatment, based on treatment protocol, were collected from the medical record. For each patient, HPV16 ctDNA counts/mL were plotted over time along with treatment histories. Percent change in HPV16 ctDNA level was calculated relative to a patient’s own baseline ((time point level - baseline level)/baseline level) and assessed at two clinical episodes. The HPV16 ctDNA level closest to the radiographic assessment was chosen to test association of HPV16 ctDNA with synchronous imaging results whereas the level on day 1 of cycle 2 of therapy was chosen to explore the ability of HPV16 ctDNA to predict radiographic progression before the standard timeline of assessment. Percent change in HPV16 ctDNA across radiographic response groups were compared with nonparametric Wilcoxon, Kruskal-Wallis, and median one-way analysis tests. We then performed ROC curve analysis for the prediction of progressive disease at time of imaging. From the ROC curve, we abstracted area under the curves (AUC) as well as the optimal cutpoint using Youden index to maximize the sum of sensitivity and specificity for predicting progression. The optimal cutpoint was estimated from the full sample and in *n* = 500 bootstrapped samples. Statistical analysis was performed using SPSS (v27) and R packages, ggplot2 (v3.2.1), cutpointR(v1.1.0).

## Abbreviations

HPV+: human papillomavirus related
OPSCC: oropharyngeal squamous cell carcinoma
ddPCR: droplet digital PCR
ctDNA: circulating tumor DNA
HNSCC: Head and neck squamous cell carcinomas
CV: coefficient of variation
